# Spectral dynamics on saturable absorber in mode-locking with time stretch spectroscopy

**DOI:** 10.1038/s41598-020-71342-x

**Published:** 2020-09-02

**Authors:** Masayuki Suzuki, Ozdal Boyraz, Hossein Asghari, Bahram Jalali

**Affiliations:** 1 Faculty of Science and Engineering, Doshisha University, 3 Tatara-Miyakodani, Kyotanabe, Kyoto 610-0394 Japan; 2 Department of Electrical Engineering and Computer Science, University of California, Irvine, CA 92697 USA; 3 Department of Electrical Engineering and Computer Science, Loyola Marymount University, Los Angeles, CA 90045 USA; 4 Department of Electrical and Computer Engineering, University of California, Los Angeles, CA 90095 USA

**Keywords:** Lasers, LEDs and light sources, Optical physics, Solitons, Ultrafast photonics, Optics and photonics

## Abstract

A mode-locked laser that can produce a broadband spectrum and ultrashort pulse has been applied for many applications in an extensive range of scientific fields. To obtain stable mode-locking during a long time alignment-free, a semiconductor saturable absorber is one of the most suitable devices. Dynamics from noise to a stable mode-locking state in the spectral-domain are known as complex and a non-repetitive phenomenon with the time scale from nanoseconds to milliseconds. Thus, a conventional spectrometer, which is composed of a grating and line sensor, cannot capture the spectral behavior from noise to stable mode-locking. As a powerful spectral measurement technique, a time-stretch dispersive Fourier transformation (TS-DFT) has been recently used to enable a successive single-shot spectral measurement over a couple of milliseconds time span. Here, we experimentally demonstrate real-time spectral evolution of femtosecond pulse build-up in a homemade passive mode-locked Yb fiber laser with a semiconductor saturable absorber mirror using TS-DFT. Capturing 700 consecutive spectra (~ 17 µs time window) in real-time using the time-stretch technique, we are able to resolve the transient dynamics that lead to stable mode-locking. Before setting stable mode-locking, an oscillating or shifting fringe pattern in the consecutive spectra was detected. This signature proves the existence of multiple pulses (including a soliton molecule) which is temporally separated with a different relative phase. The dynamics on multiple pulses is originated from a fast relaxation time of the saturable absorption effect. This study provides novel insights into understanding the pulse behavior during the birth of an ultrafast mode-locked laser pulse and the stable single-pulse operation which is highly stabilized.

## Introduction

Mode-locked lasers have been utilized as an ultrashort broadband pulse source for widespread applications, such as biomedical imaging, high precision frequency metrology, and optical spectroscopy. Due to its compact size and longtime thermal and mechanical stability semiconductor saturable absorber mirror (SESAM), it is one of the most commonly used devices that facilitate mode-locking^1,2^. Theoretically, the nature of transition dynamics from CW operation to mode-locking via quantum noise with the SESAM is well-established in past studies^1,2^. Experimentally, the behavior on spectral transition between them have not been fully understood. To fully capture the transient dynamics of mode-locking in time and spectral domains, it is necessary to measure a successive single-shot spectrum in real time with a long record length. However, a readout speed of a conventional spectrometer which consists of a grating and line sensor is several orders slower than the transient dynamics of a mode-locking process.

Recent progress on analog-to-digital converter data acquisition, a single-shot spectrum of an ultrafast phenomena can be measured in real time^3–5^. This powerful technique of single-shot spectrum measurement based on a time-stretch dispersive Fourier transformation (TS-DFT) has captured the real time spectral-temporal evolution of femtosecond mode-locking in Ti:sapphire laser^6,7^ and soliton instability in fiber oscillator^8^. In particular, the ultrafast fiber laser oscillator can intrinsically generate a multi-soliton and dissipative solitons^9–15^. To date, by using TS-DFT, several interesting soliton dynamics including build-up femtosecond laser pulses during the mode-locking have been reported mainly on the fiber laser oscillators with a nonlinear polarization rotation (NPR) or NPR assisted saturable absorbers (carbon nanotube, graphene, and so on)^16–23^. Moreover, the dynamics of harmonic mode-locking or Q-switch mode-locking have been reported on an Er fiber laser with a saturable absorber at the wavelength of 1,550 nm^21,22^. These past studies^18,21,22^ show that the saturable absorber played a role in an initial pulse generation as a starter, and therefore the dynamics on a build-up pulse in mode-locking would depend mainly on NPR. Thus, the investigation on build-up pulse formation by only SESAM (~ 500 fs) itself via single-shot spectral measurement would bring us to a novel insight into understanding the pulse formation in mode-locking, and a highly stabilized single pulse operation.

Here, we present the experimental observations of a single-shot spectrum dynamics in a homemade all polarization-maintained (PM) mode-locking Yb fiber laser with the saturable absorber mirror (SAM) by using TS-DFT. By controlling the net group delay dispersion (GDD) in the oscillator, the operation mode of our oscillator could be changed from a stable mode-locking to soliton molecule. At the cavity dispersion of 0.0024 ps^2^ and 0.034 ps^2^, the operation mode included stable mode-locking and soliton molecules, respectively. Moreover, a sliding fringe pattern was observed before reaching stable mode-locking or soliton molecules. In the stable mode-locking operation, temporally separated pulses with different relative phases appeared, but SAM lets only one of the higher intensity pulses to survive in the end. In the soliton molecule, an oscillating and sliding fringe pattern was observed. In both cases, when the observed motions of multiple pulses in which temporal separation is shorter than the relaxation time of SAM, multiple pulses were reflected by SAM. Consequently, the observed motions of multiple pulses are believed to be caused by the length of the relaxation time of the saturable absorber that has a longer the pulse duration. These experimental observations help in the understanding of the pulse formation dynamics in laser mode-locking and the optimizing of the highly stable single pulse operation.

## Results

To study the dynamics of pulse build-up with the saturable absorber itself without NPR, we have developed all PM Yb fiber femtosecond laser which consists of SAM and a dispersion management reflector (DMR). The output power was measured to be 7 mW with a pulse duration of 300 fs at the repetition rate of ~ 40 MHz. The laser pulse was extracted using a PM-tap coupler of 30% output. After propagating ~ 3 m lengths of PMF equipped with Faraday isolator, the pulse duration was measured to be 1.3 ps at the free space output. To avoid spectral distortion by the nonlinear effects in TS-DFT fiber, the laser pulse energy was attenuated by using a neutral density (ND) filter before the output light coupled into a fiber with a collimator. A detailed description of the laser set up is provided in the “Methods” section.

**Figure 1:**
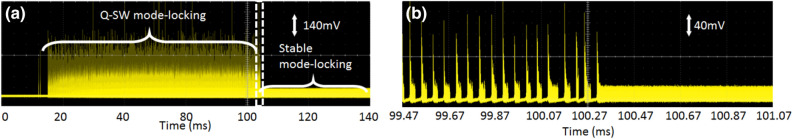
(**a**) Transient spectral dynamics of mode-locking. (**b**) Zoom of (**a**) in the time scale of 99.47–101.07 ms.

Figure 1 illustrates the transient spectral dynamics of mode-locking by using the TS-DFT. To observe the transient dynamics of mode-locking, the signal waveform was recorded over a 140 ms time window as shown in Fig. 1(a). As can be seen in Fig. 1, Q-switch (Q-SW) mode-locking before CW (stable) mode-locking was observed over a ~ 100 ms time window. In Fig. 1(a), the two short peaks at the time scale of 18 ms were unstable during Q-SW mode-locking. The unstable Q-SW mode-locking was occasionally observed just before approaching the setting power of pump LD because the pump’s LD power slowly rose to the set power. When capturing the full scale of the mode-locked transient spectral dynamics, we were not measuring the exact trigger position. To capture the full scale of the transient dynamics in edge trigger mode, the peak of the TS-DFT signal without pump LD power modulating was used. In this case, the temporal resolution was limited. Figure 1(b) shows the expanded view of the pulse formation dynamics between Q-SW and stable mode-locking. Before reaching stable mode-locking, an unstable bunched Q-SW pulses were observed and a repetition rate of couple of tens of kHz, which was independent of the oscillator cavity length. In contrast, the repetition rate of stable mode-locking corresponds to the fundamental longitudinal mode determined by the cavity length. The observed behavior of Q-SW and stable mode-locking matches the same tendency that was reported previously^8^.

**Figure 2:**
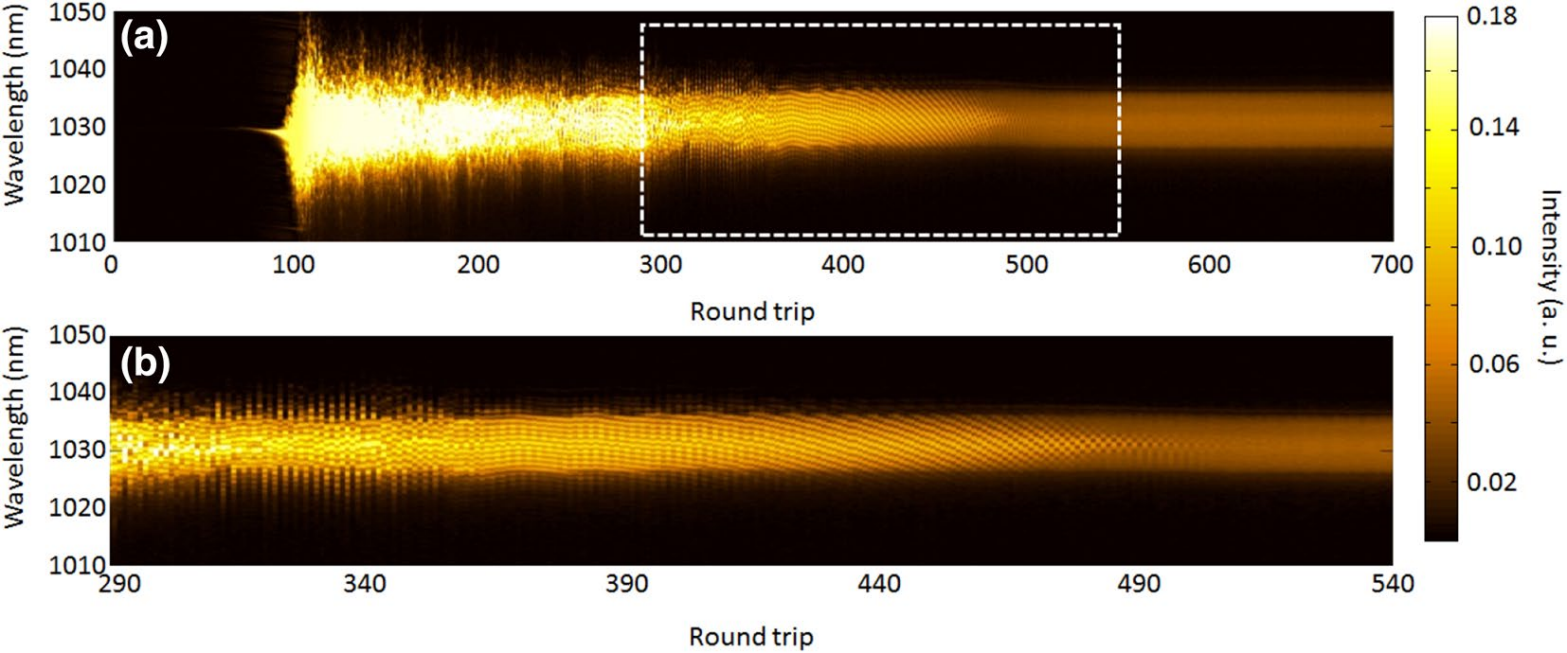
Single-shot spectra of 700 consecutive pulses between narrow spectral band pulse formation and stable mode-locking at the net GDD of 0.0024 ps^2^. (**b**) Zoom of (**a**) in the roundtrip number of 290–540.

**Figure 3:**
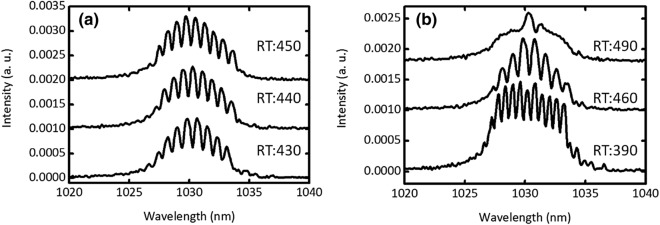
Shot-to-shot spectra at the roundtrip of (**a**) 430, 440, and 450 and (**b**) 390, 460, and 490.

Figure 2 shows the 700 (~ 17 μs) consecutive single-shot spectra of laser pulses. To capture the single-shot spectrum in a stable mode-locking regime, the detection bandwidth of the oscilloscope was set to 12.5 GHz. The net of GDD in our oscillator was set to be 0.0024 ps^2^. During the initial stage between 0 and 100 roundtrips, the energy of the narrow bandwidth pulse spectrum gradually increased. When the pulse energy approached the threshold energy of the self-phase modulation (SPM), the spectral bandwidth was suddenly broadened by SPM. From the 100th roundtrip to the 270th roundtrip, the shot–shot spectral structure was completely different from the noise-like pulse operation, which modulated simultaneously while increasing the roundtrip number and decreasing the pulse energy gradually. In past studies^17,18^, similar modulation instabilities were observed in the initial stage of pulse formation before and during stable mode-locking^8,16,17^. From 280 to 490th roundtrips, the sliding fringe was observed in the spectra. These structures of the fringe patterns in the single-shot spectrum indicate the existence of multiple pulses. Figure 3 illustrates the spectra measured after several numbers of roundtrips. As can be seen in Fig. 3(a), the fringe peak gradually shifts toward the short wavelength side while maintaining the fringe period. This tendency indicates the existence of multiple pulses with a different relative phase. Moreover, the fringe period has gradually broadened by the increasing number of roundtrips as can be seen in Fig. 3(b). The temporal period between multiple pulses was approached toward the stable mode-locking operation. Remarkably, the short and long wavelengths components disappeared, and the central wavelength fringe remained before settling at the stable mode-locked operation.

**Figure 4:**
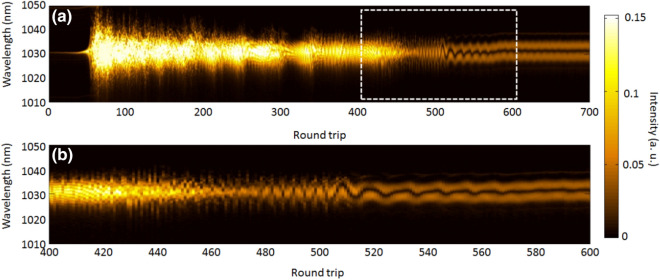
(**a**) Build-up stable soliton pair operation at the net GDD of 0.034 ps^2^. (**b**) Zoom of (**a**) in the roundtrip number of 400–600.

**Figure 5:**
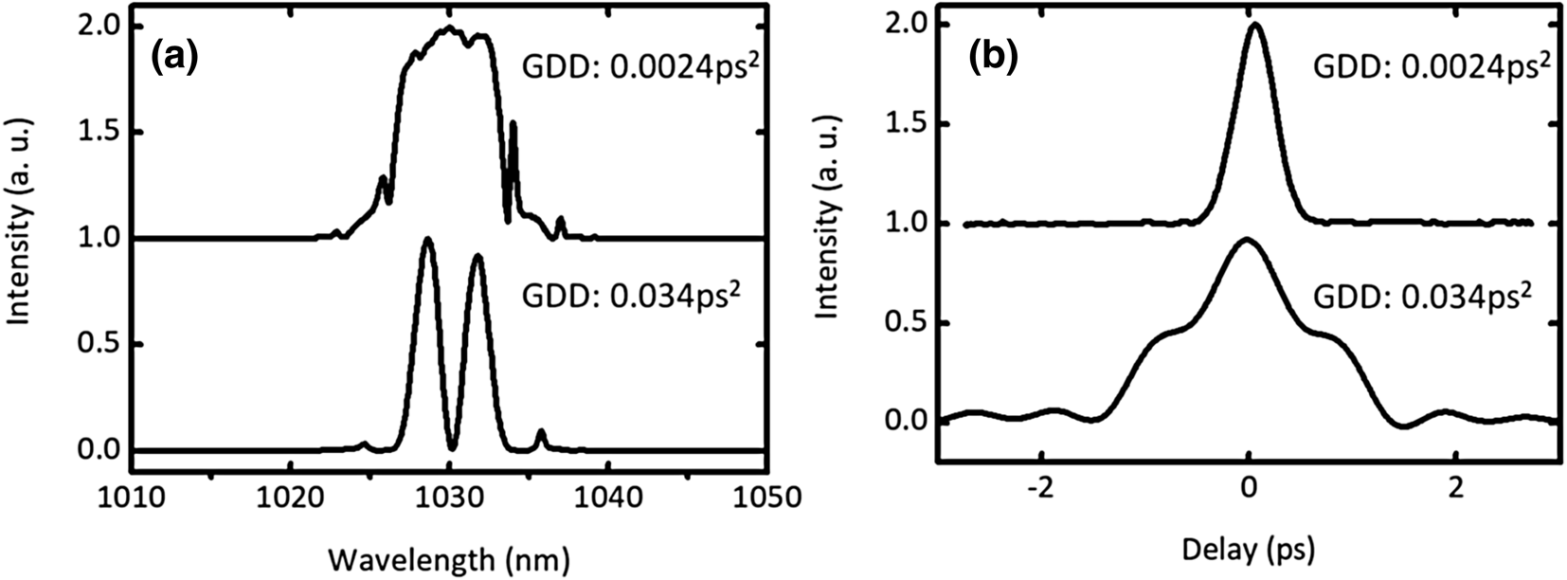
(**a**) Output spectra and (**b**) autocorrelation traces at the net GDD of 0.0024 ps^2^ (upper) and 0.034 ps^2^ (bottom).

Next, we investigated the effect of net GDD on transient dynamics of mode-locking by changing the length of the PMF between SAM and PM-WDM. Figure 4 indicates the 2D color plot image of 700 consecutive pulses at the net GDD of 0.034 ps^2^. In the initial stage of pulse build-up from a narrow spectrum to a modulation instability, the behavior of the spectral evolution is similar to the case with net GDD of 0.0024 ps^2^. It is interesting to note that we observed the oscillating two spectral peaks during ~ 100 roundtrip and then reaching to a steady state with two fixed peaks at the final stage. Figure 5 shows the time integrated spectra and autocorrelation traces at a stable steady state with a net GDD of 0.0024 ps^2^, and 0.034 ps^2^, respectively. As can be seen in Fig. 5(a), the spectral structures are completely different from each other. Notably, there are two peaks in the spectrum at 1,025 nm and 1,035 nm when the net GDD is set to 0.034 ps^2^. This spectral structure is a typical feature of soliton molecule, which is reported in previously^24^. These two solitons have a different relative phase with a slightly different temporal separation between them. The temporally close peaks were observed in the autocorrelation trace of Fig. 5(b). Thereby, the results of spectrum and autocorrelation trace support the existence of the soliton molecule. Our oscillator configuration was a dispersion management soliton (stretched pulse) with a slightly normal dispersion (close to zero) which uses a dispersion management reflector. Therefore, the soliton molecule would occur from oscillating soliton tails via a small component of Kelly sidebands when the net of GDD is slightly normal with a dispersion management soliton configuration^25^.

In our oscillator, the soliton molecule was not obtained by increasing the pump LD power. When the pump LD power was set to ~ 100 mW, the operation mode was changed from stable mode-locking to noise-like pulse generation. In addition, the noise-like pulse was not self-started in our oscillator. Consequently, the soliton molecule was only obtained when the length of the cavity was extended in our study.

## Discussion

We attribute the observation of the fringe pattern shifting before stable mode-locking to the changing of the relative phase between two pulses. When the net GDD was set to 0.0024 ps^2^, the surviving two pulses, which were observed just before stable mode-locking, were due to the longer relaxation time of saturable absorption in comparison to the pulse width. The SAM relaxation time was 500 fs, which is quite longer than ~ 110 fs separation between two pulses. This separation is calculated from the fringes that exists at the central wavelength of 1,030 nm and when the roundtrip number is ~ 490. The transparency, and hence reflectivity of saturable absorber mirror, increases with the rise of the pulse energy. Therefore, the high-intensity pulse survives and the others are suppressed in SAM when the high-intensity pulse is the trailing in time and low-intensity pulse arrives before SAM. Simultaneously, the low intensity pulse components at the short and long wavelengths are absorbed by SAM, besides being unamplified by the YDF. In particular, the reflectivity of SAM during ~ 500 fs pulse duration was quite high since it is already saturated. On the other hand, the reflectivity of SAM drops exponentially with a ~ 30 ps time constant during the absorption recovery^26^. Consequently, one of the two pulses, the higher intensity single pulse survives as the stable mode-locking operation during the fast relaxation time when the roundtrip number is ~ 500 or more. This experimental behavior is the first observation of spectral formation in SAM itself and is supported by the theoretical pulse formation architecture in the saturable absorber^2^.

In contrast, when the net of GDD is set to 0.034 ps^2^, a stable soliton molecule was observed as seen in Fig. 5. The soliton molecule results from the increasing pulse energy by increasing the length of the fiber between WDM and SAM in the cavity. In our study, the output power nominally stayed constant at ~ 7 mW when the cavity length changed. Extending the cavity length increased the pulse energy, and therefore the pulse breaks up into two pulses with half energy. When the separation time between the two pulses is within the relaxation time of SAM, the two pulses are reflected by SAM. Consequently, phase-locked soliton pairs appear at the temporal separation shorter that the SAM relaxation time of 500 fs as shown in Fig. 5(a, b) (bottom solid carves). This feature is a typical behavior of a saturable absorber that was predicted theoretically in past studies^27,28^. As it can be seen in Fig. 4, the oscillatory of spectral fringes were observed with increasing roundtrip numbers just before reaching a stable soliton molecule (bound state). The signature of vibrating fringe pattern is due to the oscillating phase between two different temporal separation pulses. With increasing the roundtrip for the stable soliton molecule, the relative phase of two temporal separation pulses evolves to the ~ 560th roundtrip and finally locks it at the ~ 600th roundtrip. In past studies^13,18^, the oscillating soliton molecules were observed in the unstable transition in mode-locking operation, or before setting stable mode-locking in the build-up pulse formation. Our experimental observation shows the same tendency that was reported previously in the pulse build-up experiments with the Er fiber laser^17,18^.

These dynamics of transient mode-locking or soliton molecule are composed of the build-up of narrow spectral bandwidth pulses, the modulation instability, and the dissipative soliton motion. These transient dynamics on the stable mode-locking operation or soliton molecule are common. This behavior was observed in every measurement and each duration, however, it was random. Thus, the dynamics of pulse formation in both net GDDs have reproducibility. The soliton molecule was observed in the initial stage of pulse formation when the cavity dispersion is anomalous and has a slightly normal dispersion^17,18,25^. In contrast, no soliton molecule was observed in the transient dynamics between CW and stable mode-locking in the past study^29^. Thereby, the formation of soliton molecule in the initial stage of pulse formation would be the universal dynamics in the net GDD of anomalous and slightly normal dispersion.

To understand the periodic soliton molecule dynamics, the relative phase was reconstructed by an absolute wavelength calibrated TS-DFT measurement in the past study^7^. A precise determination of wavelength is necessary to retrieve the phase of the single-shot spectrum^20^. In transient dynamics on initial pulse formation, the temporal waveform might be shifted by the synchronized jittering. In our study, we have calibrated our wavelength axis by matching to OSA measurement spectrum at the stable mode-locking operation in the final stage of transient dynamics. To investigate the phase analysis, the precise spectral measurement with the compensation of the temporal shifting of the waveform in the transient dynamics is required. We plan to develop the absolute wavelength calibrated TS-DFT and analyze the phase of the single-shot spectrum in future work.

Another potential solution is the time stretch spectral shear interferometry^5^. Normally, interferometry only measures the relative phase of the two paths, not the absolute phase of the laser pulse. However, because of the interferometry delay, the stationary frequency (wavelength) is different for the two arms. The interferometer is linear and does not change the optical frequency, however, a frequency shear is created due to the combination of the interferometer and frequency to time mapping^30^. If a sufficiently large shear frequency can be created, this technique can characterize the amplitude and phase of laser pulse directly.

It is a well-known fact that the Fourier transform spectrum corresponds to field autocorrelation as reported in past studies^7,17,21,22^. No phase information of shot-to-shot spectrum measurements were obtained in our study, and therefore the reconstruction of field autocorrelation could not be performed. To accomplish the reconstruction of an autocorrelation pulse, we will need to measure the phase of the shot-to-shot spectrum in our future work.

To understand the pulse evolution on build-up pulses from quantum noise to steady state mode-locking, numerical studies have been conducted by using a Nonlinear Schrödinger equation (NLSE) or complex Ginzburg–Landau equation (CGLE)^31,32^. However, the dynamics of multiple pulse behavior observed in our study were not captured in these numerical studies. Therefore, we believe these experimental observations will have an impact on future numerical studies of spectral dynamics from noise to stable mode-locking.

In conclusion, we reported on the spectral dynamics of the pulse formation in mode-locking with the homemade PM fiber laser with SAM using TS-DFT. The pulse formation behavior on the saturable absorber have been directly shown by measuring the real-time single-shot spectrum. When the net GDD was set to 0.0024 ps^2^, the temporal separation pulse with a different phase appeared before settling on the stable mode-locking, and then the dynamics of pulse formation, which the single pulse survived from the multiple pulse by saturable absorber, was observed. When the net of GDD was set to 0.034 ps^2^, the oscillating and shifting motion of the soliton molecule appeared as before with the stable soliton molecule. The experimental observations are the typical architecture of theoretical saturable absorption effects. We believe that these results open new opportunities to the understanding of femtosecond pulse formation and the stabilizing of laser pulse.

## Methods

**Figure 6:**
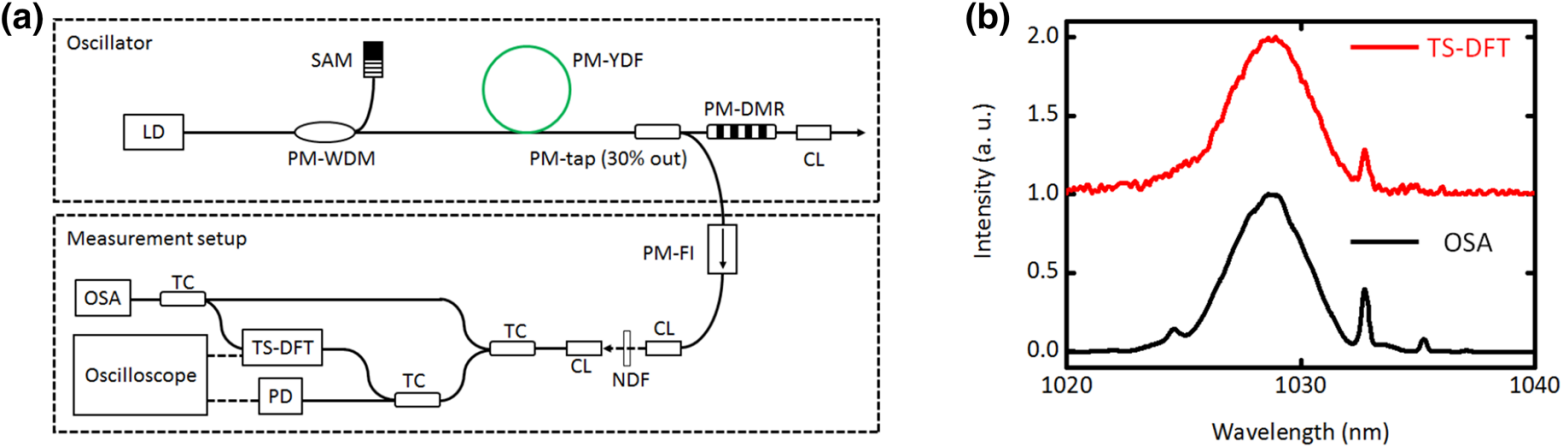
(**a**) Schematic layout of the Yb fiber laser and real time spectral measurement setup. LD, laser diode; PM-WDM, polarization maintain wave division multiplexer; PM-YDF, polarization-maintaining Yb-doped fiber; PM-TC, polarization-maintaining tap coupler; PM-DMR, polarization-maintaining dispersion management reflector; CL, collimation lens; PM-FI, polarization-maintaining Faraday isolator; NDF, neutral density filter; TC, tap coupler. (**b**) The spectra of mode-locked laser pulse from TS-DFT and OSA (without TS-DFT).

Figure 6(a) shows the experimental setup of our study. A homemade oscillator consists of a pump LD at 976 nm, a PMF, a PM wavelength division multiplexer (PM-WDM), a SAM, a PM Yb-doped fiber (PM-YDF), a PM tap coupler (PM-TC) (30% out), and a PM dispersion management reflector (PM-DMR). The length of PMF and YDF are 2.0 m (or 2.2 m) and 0.4 m, respectively. The total optical fiber length matches the laser repetition rate of ~ 40 MHz. The large dispersion of PMF was compensated by the PM-DMR (DMR-1030-20-12(+D0.2+0)-P, TeraXion). The reflection of PM-DMR is to be 80% with a 3-dB spectral bandwidth of 20 nm at a wavelength of 1,030 nm. To change net of GDD, the length of PMF between PM-WDM and SAM was changed. The mode-locking was initiated from SAM (SAM-1030-52-500 fs-FC/APC-PM980, Batop) by increasing pump LD power. The mode-locked was self-started by increasing the pump LD power up to 66 mW. The output power and pulse duration at the free space are 7 mW and 1.3 ps, respectively. The pulse duration was recompressed from 1.3 ps to 300 fs by using the grating pair. By extending the pulse energy of 0.6 nJ/pulse and by calculating the output power and repetition rate, the operation mode changed from the stable mode-locking to the soliton molecule.

A single-shot spectrum was measured by using TS-DFT. The details of TS-DFT were described elsewhere^5^. Briefly, the temporal stretched signal was recorded by real time oscilloscope (DSA 71254C, Tektronix) with an analog bandwidth of 12.5 GHz, and 100 GSa/s sampling rate. The dispersion at the wavelength of 1,030 nm is calculated to be − 530.5 ps/nm. The length of fiber is ~ 4.2 km corresponding to the pulse duration after stretching of ~ 2 ns. Simultaneously, the shot-to-shot pulse energy was measured by using a photodetector (DET08CFC/M, Thorlabs) with the oscilloscope. To check absolute spectrum of TS-DFT, we have measured the spectrum by using optical spectrum analyzer (OSA) (AQ6370C, Yokogawa Electric Corporation). The spectral resolution of TS-DFT was estimated to be 0.07 nm in our case. To avoid the spectral changing in TS-DFT, the input laser pulse energy was attenuated by using the ND filter (NDF). The spectrum of TS-DFT is reasonable agreement with that of OSA (without TS-DFT) as shown in Fig. 1(b).

When one turns on the pump LD, the output power of the pump LD gradually increases. Before approaching the set pump LD power of 66 mW, Q-SW mode-locked was observed as shown in Fig. 6(b). The period of the Q-SW mode-locked region was different in the experiment, and therefore the capture timing (trigger) of the TS-DFT signal was unstable. To stabilize the trigger, the pump LD power has modulated with the threshold of the Q-SW operation pump LD set to ~ 50 mW.
